# In Vivo Toxicity and In Vitro Solubility Assessment of Pre-Treated Struvite as a Potential Alternative Phosphorus Source in Animal Feed

**DOI:** 10.3390/ani9100785

**Published:** 2019-10-11

**Authors:** Soomin Shim, Seunggun Won, Arif Reza, Seungsoo Kim, Sungil Ahn, Baedong Jung, Byungil Yoon, Changsix Ra

**Affiliations:** 1 Department of Animal Industry Convergence, College of Animal Life Sciences, Kangwon National University, Chuncheon 24341, Korea; vibrato7@naver.com (S.S.); reza.arif@kangwon.ac.kr (A.R.); seung_su89@nate.com (S.K.); sorzente@naver.com (S.A.); 2 Department of Animal Resources, College of Life and Environmental Science, Daegu University, Gyeongsan 38453, Korea; swon@daegu.ac.kr; 3 Department of Environmental Science, College of Agricultural Sciences, IUBAT—International University of Business Agriculture and Technology, Dhaka 1230, Bangladesh; 4 Department of Veterinary Science, Kangwon National University, Chuncheon 24341, Korea; bdjung@kangwon.ac.kr (B.J.); byoon@kangwon.ac.kr (B.Y.)

**Keywords:** phosphorus, struvite, swine wastewater, pre-treatment, toxicity, solubility, animal feed

## Abstract

**Simple Summary:**

Phosphorus (P) is an indispensable element needed for the growth and development of all living organisms. Phosphorus is mined primarily from non-renewable phosphate rock reserves. The use of P as fertilizer and feed additives continues to rise with the increasing global population. We therefore need to find an alternative and renewable, as well as sustainable, source of P to fulfill the demands of agriculture and livestock. Phosphorus recovered as struvite from livestock wastewater could be a sustainable alternative to commercial P sources, while its application has only been limited to arable lands as fertilizer. This study elucidates that struvite can also be used as an alternative P source in animal feed other than fertilizer through proper pre-treatment. Phosphorus recovery from livestock wastewater and its reutilization as an animal feed ingredient would therefore be a good strategy to substitute commercial P sources and ensure societal sustainability.

**Abstract:**

Apart from using as fertilizer for plants, the application of struvite may be expanded to animal feed industries through proper pre-treatment. This study aimed to investigate the safety and efficacy of using pre-treated struvite (microwave irradiated struvite (MS) and incinerated struvite (IS)) in animal feeds. For safety assessment, an in vivo toxicity experiment using thirty female Sprague Dawley rats (average body weight (BW) of 200 ± 10 g) was conducted. The rats were randomly divided into five groups, including a control. Based on the BW, MS and IS were applied daily by oral administration with 1 and 10 mg kg^−1^-BW (MS1 and MS10; IS1 and IS10) using dimethyl sulfoxide (DMSO) as a vehicle. A series of jar tests were conducted for four hours to check the solubility of the MS and IS at different pH (pH 2, 4, 5, 6 and 7) and compared to a commercial P source (monocalcium phosphate, MCP, control). The toxicity experiment results showed no significant differences among the treatments in BW and organ (liver, kidney, heart, and lung) weight of rats (*p* > 0.05). There were no adverse effects on blood parameters and the histopathological examination showed no inflammation in the organ tissues in MS and IS treated groups compared to the control. In an in vitro solubility test, no significant difference was observed in ortho-phosphate (O-P) solubility from the MCP and MS at pH 2 and 4 (*p* > 0.05), while O-P solubility from MS at pH 5 to 7 was higher than MCP and found to be significantly different (*p* < 0.05). O-P solubility from IS was the lowest among the treatments and significantly different from MCP and MS in all the experiments (*p* < 0.05). The results of this study not only suggest that the struvite pre-treated as MS could be a potential alternative source of P in animal feed but also motivate further studies with more stringent designs to better examine the potential of struvite application in diverse fields.

## 1. Introduction

Phosphorus (P) is a key element for animal and plant growth as well as an important nutrient for future food security. It has been estimated that the present reserve of phosphate rock is 70,000 million tons as P_2_O_5_, of which 40 million tons of phosphate rock is mined every year [1,2]. Studies reported that the resource may be exhausted within the next 90–300 years, with an expected annual increase in P consumption of 2% [1,3]. Phosphate rock was recently categorized as one of the 20 critical raw materials in Europe since P utilization has been known as a one-way flow and it is classified as a non-renewable resource. In the world, it is mainly three countries (Morocco, China, and the United States), which take the lead in intensive production for phosphate rock [4]. In addition, due to non-replaceability and high economic importance, there might be a political and social tension built over the phosphate rock reserves and the world could move from an oil-based to a phosphate-based economy, as the aforementioned three countries control more than 85% of the known global phosphorus reserves [5]. 

The majority (80 to 90%) of the P mined from phosphate rock reserves has been used in the agricultural sector for fertilizer and animal feed production [6]. Whereas, of the total phosphate fertilizer applied in the arable lands, 57% can be lost in soil leaching, erosion, and runoff [7,8]. Moreover, livestock wastewater contains relatively high strengths of P concentration, which leads to eutrophication [9], resulting from excessive P contents inevitably given to achieve high growth performance. Every year 7 million tons of P may be released to the environment through animal manure and excreta [10]. P recovery and recycling from highly polluted livestock wastewater could therefore be a sustainable alternative to commercial P sources. 

For P recovery from different wastewater, various physicochemical techniques have been introduced. Among those methods, struvite precipitation has been widely studied with its very straightforward regime and is considered as an eco-friendly and sustainable process in terms of nutrient recovery and recycling from nutrient-rich waste streams. 

Along with economic development, swine farming practices in Korea are becoming more intensive. Intensive swine farming practices result in an increase in swine wastewater generation. Swine wastewater treatment practice in Korea is quite different compared to European and North American countries. The Korean government is currently operating more than 200 centralized swine wastewater treatment plants with a treatment capacity of more than 20,000 tonnes per day [11,12]. The bulk recovery of P in the form struvite from the centralized treatment plants might therefore be economical and sustainable. Moreover, the strategy of recovering and recycling P from swine wastewater and its reutilization as an animal feed ingredient can help protect the environment by reducing nutrient contents in discharged effluents as well as initiating a P circular economy.

Currently, the application of struvite has only been limited to arable lands as fertilizer and reported to be non-toxic to plants [13]. Moreover, P bioavailability from struvite has shown similar performance as the chemical fertilizers in crop trials [14]. Furthermore, to our knowledge, none of the studies focused on exploring the feasibility of struvite application in animal feed as an alternative P source. Swine wastewater generally contains high amounts of ortho-phosphate (O-P) and ammonium nitrogen (NH_4_-N). NH_4_-N is highly toxic for the animals and its accumulation in cells can damage the tissues [15]. It is therefore necessary to pre-treat the struvite recovered from swine wastewater for its further use in animal feed. We have already successfully tested the feasibility of reutilizing P recovered from swine wastewater as a dietary supplement in juvenile far eastern catfish (*Silurus asotus*) by comparing with a commercial P source [16,17]. In this study, we suggest manufacturing options for struvite recovered from swine wastewater to ensure biological safety and present the possibility of struvite application as an alternative P source in animal feed rations, with the results of an in vivo toxicity test on rats and an in vitro solubility test.

## 2. Materials and Methods 

### 2.1. Struvite Production from Swine Wastewater and Pre-Treatment 

Swine wastewater was collected from the storage tank of a gestation pig barn located in Chuncheon, South Korea. After collection, the solid/liquid separation was done using centrifugation at 3000 rpm for 10 min and the liquid part was used for struvite production. The reactor had an effective volume of 20 L, including a compartment of about 2 L as a reaction zone, and the rest was composed of a settling zone and recovery zone. The influent with the flow rate of 0.11 L min^−1^ (160 L d) was introduced from the top and passed through the compartment with holes at the bottom. Crystals formed in the reaction zone were precipitated and the flux flew up to the outlet, which resulted in a hydraulic retention time (HRT) of 3 h in the entire P recovery process. The precipitates were collected from the bottom where the valve was equipped. MgCl_2_ in the liquid phase was provided into the reactor to meet the appropriate Mg to P ratio (~1.3) in the influent, which was found as an optimal condition in our previous research [18]. An air diffuser was set at the bottom of the compartment in the reaction zone for CO_2_ stripping and, thus, a pH of over 8.5 was maintained. An X-ray diffraction (XRD) analysis (X’Pert PRO MPD, PANalytical B.V., Netherlands) was conducted to give the precipitates an identity, where the peaks indicate that the precipitates recovered were identified as struvite. The supernatant was put back into the reactor after collecting precipitates from the bottom of the struvite reactor. The settled materials were spread on the tray and stored in a dry-oven at 20 °C for 2 days, then the moisture content of pre-dried sample was measured at 105 °C following the standard methods [19], which resulted in a moisture content of 35%, but water parts in struvite evaporated during the second drying at 105 °C. To remove all possible toxicity, recovered precipitates were pre-treated in a microwave (80 Hz g^−1^ for 5 min) and a muffle furnace at 550 °C for over 30 min after drying [3,17]. The heavy metal contents in the pre-treated struvites were determined using inductively coupled plasma atomic emission spectroscopy (ICP-AES) (Optima 7300 DV, PerkinElmer, Waltham, MA, USA).

### 2.2. In Vivo Toxicity Assessment in Rats

An in vivo toxicity experiment using rats was conducted to evaluate the safety of using pretreated struvite (microwave irradiated struvite (MS); incinerated struvite (IS)) as an alternative to commercial P sources in animal feeds. The protocol of the experiment was approved and the rats were cared for according to the guidelines of the Institutional Animal Care and Use Committee of Kangwon National University, Chuncheon, South Korea (ACUC # KW 130620-1). For the toxicity experiment, 30 female Sprague Dawley rats (average body weight (BW) of 200 ± 10 g) were procured from Bio Genomic Inc. (Charles River Technology, Gapyung-Gun, Korea) and were allotted to each of five groups, including a control, in a randomized complete block design, resulting in six rats per treatment group. The sample size was calculated using the resource equation method [20]. All groups of rats were given ad libitum access to water and conventional laboratory diets (5L79, Labdiet, St. Louis, MO, USA) to maintain their normal physiology. The amount of crude protein, crude fat, crude fiber, ash, Ca, and P was 18, 5, 5, 8, 0.85, and 0.70%, respectively, in the supplied lab diet. To observe the toxic effects of test materials, subacute in vivo toxicity tests were conducted for 28 days [21]. Based on the observations regarding the highest dose of ammonia without effect reported by Tuleka et al., pre-treated struvite doses of 1 and 10 mg kg^−1^ BW were designated to evaluate the repeated dose toxicity [22]. MS and IS of 1 and 10 mg kg^−1^ BW were dissolved in 1% dimethyl sulfoxide (DMSO) as a vehicle, which were represented by MS1, MS10, IS1, and IS10, respectively, in 4 treatment groups. Nutrient contents of the treatments are shown in Table 1. The prepared testing materials were given daily by oral administration at once. A control group was only provided with the carrier (1% DMSO). During the experimental period, the cycle of light/dark was maintained at 12 h/12 h, respectively, and the temperature of the experimental cages was maintained at 20–25 °C. The feed intake and body weight were recorded regularly. Rats were monitored for any abnormalities in activities, behavior, and general health status, and fed with respective experimental diets. 

At the end of the experimental feeding (28 d), the final BW was measured and all the rats were euthanized and sacrificed. Blood samples of 5 mL were collected from all rats by heart puncture using the sterilized needles and syringes in a disposable vacutainer tube without any anticoagulant and stored at 4 °C for 2 h. After centrifugation (3000 rpm for 10 min at 4 °C), serum samples were separated and stored at −20 °C in Eppendorf tubes. Internal organs (liver, kidney, lung, and heart) were removed and weighed from all rats after they were sacrificed. For histopathological assay of the internal organs, the tissue samples of heart, liver, kidney, and lungs were collected and fixed in 10% neutral buffered formalin, embedded in paraffin wax, and cut into 5 µm-thin sections. For routine examination, all samples were stained with hematoxylin and eosin (H&E) and observed under an optical microscope (×100 or ×400) [23]. The blood tests were conducted at the end of the experimental feeding; i.e., after 24 h from the last dose of testing materials. Assay kits for the analysis of serum biochemicals were obtained from Chemon Inc., South Korea. Serum biochemical parameters including alanine aminotransferase (ALT), aspartate aminotransferase (AST), blood urea nitrogen (BUN), creatinine (CRE), inorganic P (P_i_), and calcium (Ca) were examined to assess the effect of the treatments on the internal organs [24,25]. The selected serum biochemical parameters were analyzed using an automated blood biochemical analyzer (Model AU400, Olympus, Tokyo, Japan) and an automated electrolyte analyzer (Model M744 (Na^+^/K^+^/Cl^−^) analyzer, Siemens, Deerfield, MA, USA).

### 2.3. In Vitro Solubility Test

A series of jar tests were conducted for four hours at 25 °C to check the solubility of the test materials by simulating the gastrointestinal pH conditions (pH 2, 4, 5, 6, and 7). The required amount of test materials was calculated based on the total P content of the materials. A total of 0.5 g (particle size < 100µm) of each of the test materials were weighed exactly in a 1L beaker. The beakers were filled with de-ionized water. The pH of the solution was monitored constantly and the necessary pH adjustment was done with 1M HCl throughout the experiment. All the experiments were performed in triplicate. Considering the variation of gastrointestinal transit time among animals, immediate release of P at low pH, and the availability for absorption, 2 mL of sample was taken per treatment at 15, 30, 60, 90, 120, 150, 180, 210, and 240 min intervals, diluted with 18 mL de-ionized water, centrifuged at 3000 rpm for 10 min, and O-P content was determined using an auto-analyzer (Quik Chem 8500, LACHAT, Loveland, CO, USA).

### 2.4. Statistical Analysis

Statistical analysis was conducted using GraphPad Prism (version 5.03, 2009). Data obtained from the in vivo toxicity test were analyzed with the one-way analysis of variance (ANOVA) test, considering treatments as a fixed factor. Multiple comparisons were performed using Tukey’s HSD tests. In vitro solubility of O-P was analyzed by comparing the O-P concentrations at the beginning with those at the end of the experiment (240 min) using an ANOVA with O-P concentrations at 9 time points as a repeated measure. A *p*-value of < 0.05 was designated to determine statistical significance.

## 3. Results and Discussion

### 3.1. P Recovery and Manufacturing 

**Figure 1:**
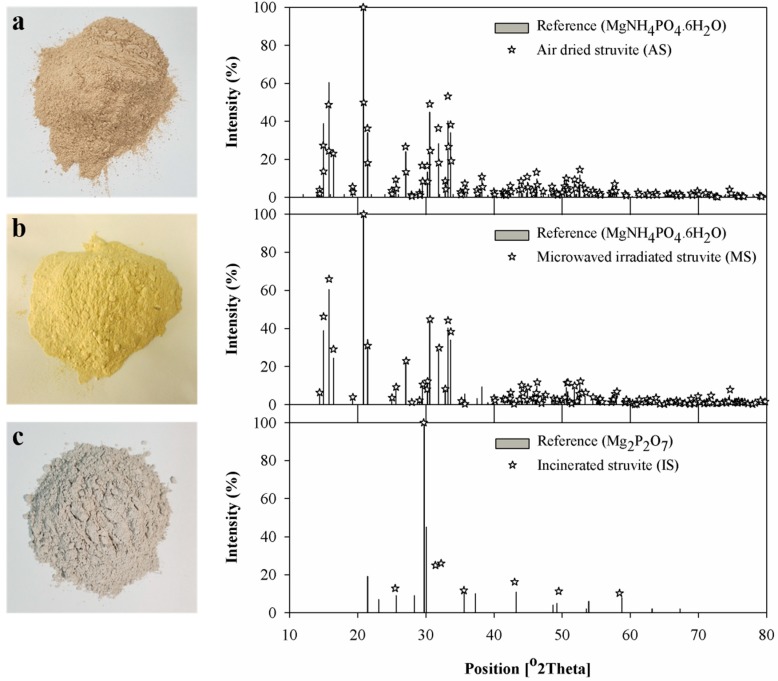
Pictures of recovered and pre-treated struvites with the identification by XRD analyses. (**a**) Air-dried at 20 °C for 2 days, (**b**) microwave irradiated at 80 Hz g^−1^ for 5 min, and (**c**) incinerated at 550 °C for 30 min (* Asterisks in each graph indicate reference materials from XRD analyses).

Using a lab-scale reactor (160 L d^−1^), an O-P removal efficiency of 93.1% was observed and the precipitate production rate and yield of 8.7 ± 0.6 kg struvite m^−3^_reactor_.d^−1^ and 9.8 ± 0.5 g struvite g^−1^O-P_input_, respectively, were achieved. The recovered precipitates were identified by XRD analysis, which indicated the recovered material was struvite (Figure 1a). The precipitates contained a P content of 21.6%, which was higher over the theoretical P of 12.7% in struvite since the P in the form of organic compounds might be present in the precipitates. 

Different from the struvite recovered from municipal and industrial wastewater, struvite recovered from livestock wastewater is relatively safer for its application to agricultural land [13] because all the ingredients in precipitated struvite from livestock wastewater originate from animal feedstock and include relatively lower amounts of harmful metals. Instead of phosphate rock mined from P reserves, recovered P via struvite precipitation contains lower heavy metal content and needs lesser efforts to purify for target use, such as for fertilizer and chemical industries [26]. During this study, the trace heavy metals in the recovered struvite were rarely found or not detected and satisfied the highest standard limits of feedstock for animal growth as suggested by the Korean Regulatory Authority (Table 2) [27]. In addition to the use in fertilizer sector, struvite as a way of P recycling can therefore be applied in the animal feed industry like phosphate rock. However, it is very important to ensure that the heavy metal contents in the recovered struvite are within the safe limits prior to use as animal feed additive.

The theoretical N content is only around 5.8% in struvite, with NH_4_-N being the main N species, and further N content could be decreased through air drying (Table 2). However, N content in air-dried struvite was still higher than the typical N content in animal feeds (1.9 to 4.8%) [28]. Therefore, additional treatment is required to reduce the N content before its application in feed production. To ascertain cost-effective treatments for the reduction of the N content and the removal of organic compounds, microwave irradiation and incineration treatments were selected according to our previous study [3]. Both treatments were conducted to remove mainly NH_4_-N from struvite. Microwave irradiation treatment did not show much influence on its morphology and it was still determined as struvite with XRD analysis (Figure 1b), but incineration under 550 °C denatured struvite to magnesium pyrophosphate (Mg_2_P_2_O_7_) by removing ammonium and hydrates, which was confirmed by XRD analysis as well (Figure 1c). Ammonium and hydrate contained in struvite started to be released as a gas phase at 55 °C and completed at 250 °C due to the thermal decomposition [29]. The N concentration of 5.0% in air-dried struvite was decreased 3.713 and 0.003% through microwave irradiation and incineration treatments, respectively (Table 2). Moreover, after pre-treatment of recovered struvite with the decrease in moisture content, an increase in the P, Ca, and Mg concentrations in the pre-treated materials were observed (Table 2). Hence, if a specific amount of struvite is added to the animal feed, considering the dietary P requirement, the concentration of other minerals present in the struvite might be higher than the requirement. Further toxicological studies are therefore needed to confirm the safety and efficacy of pre-treated struvites for their use in animal feed, although pre-treatments reduce the N content in struvite. 

### 3.2. Toxicity Test in Rats

**Figure 2:**
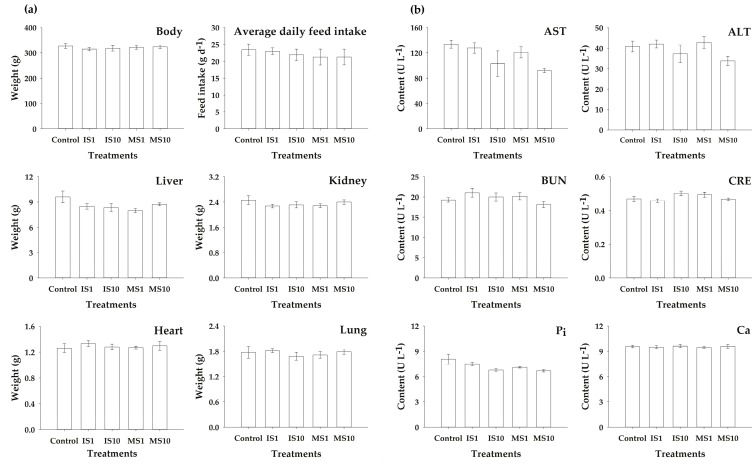
Comparison of (**a**) body and internal organ weights and (**b**) blood metabolites of rats treated with control, IS, and MS groups (Control (only 1% DMSO)); IS1 and IS10, rats fed with IS at 1 and 10 mg kg^−1^ BW; MS1 and MS10, rats fed with MS at 1 and 10mg kg^−1^ BW; Error bar: Standard error of means in each group (n = 6)).

Prior to applying struvite recovered swine wastewater as a P additive in the animal feed ration, it is necessary to conduct the toxicity test to ensure biological safety. We therefore pre-treated struvite recovered from swine wastewater as MS and IS. After 28 d of feeding MS and IS, along with control feed, to the rats, the average total BW gain of nearly 121.4 ± 20.3 g was observed in the treated groups, including the control group. However, no statistical differences were found in BW, average daily feed intake, and the internal organs (liver, kidney, heart, and lung) among all the experimental groups fed with different doses of MS and IS, including the control (*p* > 0.05) (Figure 2a).

In the administration with only the vehicle (control), the blood metabolites levels in rats were found to be normal (Figure 2b). No significant alteration in AST and ALT levels between IS1, IS10, MS1, MS10 (1 and 10 mg kg^−1^ BW), and the control groups were observed. The BUN, CRE, Pi, and Ca levels (mg dL^−1^) of blood were found within the normal range and no statistical differences (*p* > 0.05) were observed in MS and IS treated groups compared to the control. 

**Figure 3:**
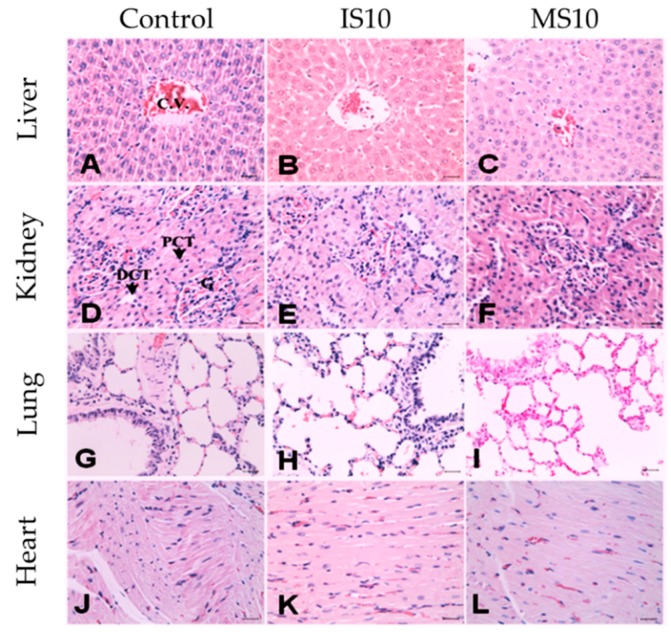
Comparison of histopathological examination of the internal organs of rats treated with control, IS, and MS groups (Control (only 1% DMSO); IS10, rats fed with IS at 10 mg kg^−1^ BW; MS10, rats fed with MS at 10 mg kg^−1^ BW.

The histopathological analyses of the treatment groups applied 10 mg kg^−1^ BW of MS and IS displayed the normal shape, size, and color of the internal organs. Tissues from each organ (liver, kidney, lung, and heart) were found to be normal and no mark of inflammation, necrosis, edema, and/or any other specific pathological symptoms were noticed at such a higher dose of struvite supplementation (Figure 3). 

Struvite contains an ammonium ion, which is thought to be toxic for cellular components for animals. Ammonia and ammonium ions are toxic to animal cells and especially influence the cell membrane [15,30] through interrupting the enzymatic reactions and intracellular pH changes, which may lead to the disturbance of proton gradients and the inhibition of endocytosis and exocytosis [31]. A higher dose of ammonium compound (300 ppm ammonium perfluorooctanoate) in rats caused an elevated liver weight, an increase in the incidence of diffuse hepatocellular hypertrophy, portal mononuclear cell infiltration, mild hepatocellular vacuolation [32], and some reproductive abnormalities in male and female rats were also found with a higher dose of ammonium compounds [33]. The present study did not find any abnormalities or lesions in the internal structures of the lungs, liver, kidney, and heart. It might be stated that, although struvite contains ammonium, it would not be harmful to animals after microwave irradiation treatment or incineration, thus ensuring animal welfare. 

The homeostatic condition of P, Ca, and Mg in the body is important as they control regular cellular activities such as energy metabolism and cell signaling [34]. Numerous cellular and tissue injuries including impaired bone mineralization, increased cell death, impaired cell signaling, vascular calcification, pre-mature aging, renal dysfunction, increases tumorigenesis, impaired fertility, skeletal development, etc., are associated with excessive retention of the above-mentioned macronutrients in the body [35,36,37,38,39,40]. In this study, no abnormalities in internal organ weights and serum biochemicals were observed. Therefore, the results obtained from growth performance, blood biochemical report, and histopathological studies of the internal organs indicated that the administration of MS and IS as a P additive retrieved from swine wastewater had no toxic effects on rats.

### 3.3. In Vitro Solubility Test

**Figure 4:**
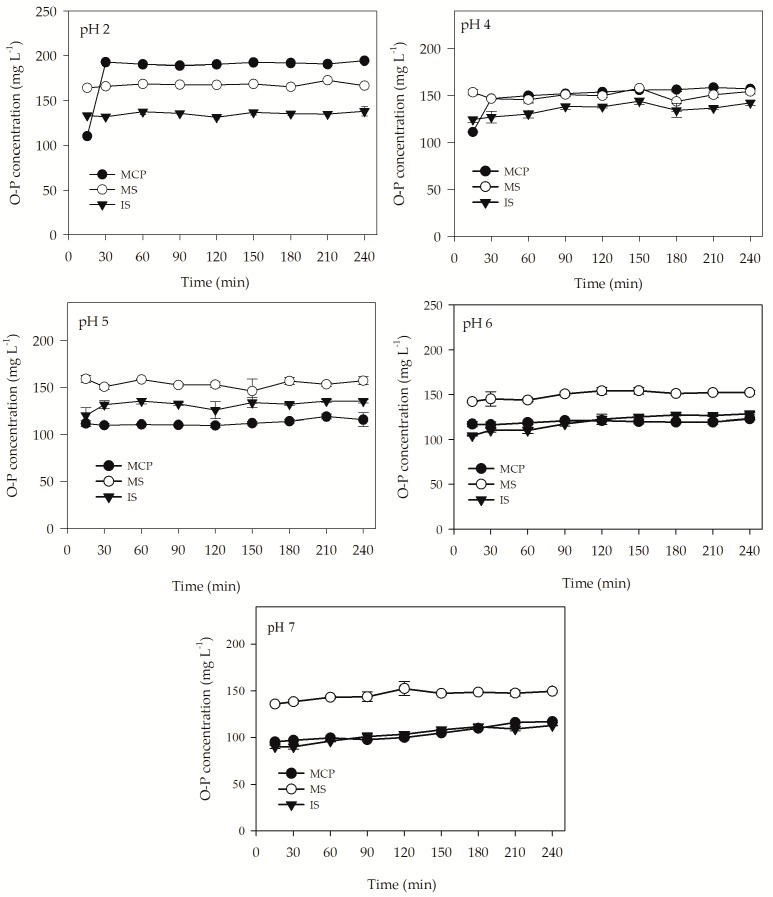
Variations in O-P concentration in an in vitro solubility test of each test materials over time.

Variations in O-P concentration of the test materials at different pH levels (pH 2, 4, and 5) during the in vitro test are shown in Figure 4. All the experiments started with a constant concentration of the test materials. At pH 2, O-P concentrations of MCP, MS, and IS varied from 110.3 to 194.5 mg L^−1^, 164.4 to 172.9 mg L^−1^, and 131.4 to 138.2 mg L^−1^, respectively. At the end of the experiment, 98.5%, 83.4%, and 69.1% of the total P in MCP, MS, and IS, respectively, was dissolved at pH 2 (Table 3). At pH 4, O-P concentrations of MCP, MS, and IS ranged from 111.3 to 158.8 mg L^−1^, 143.8 to 158.1 mg L^−1^, and 124.5 to 143.4 mg L^−1^, respectively, and it was found that at the end of the experiment 79.7%, 77.2%, and 70.4% of the total P in MCP, MS, and IS, respectively, was dissolved. At pH 5, O-P concentrations of MCP, MS, and IM were varied from 109.7 to 119 mg L^−1^, 146.1 to 159.2 mg L^−1^, and 119.9 to 135.4 mg L^−1^, respectively, and, at the end of the experiment, showed solubilities of 57.8%, 78.6%, and 67.6%, respectively. At pH 6, O-P concentrations of MCP, MS, and IS were ranged from 116.6 to 123.1 mg L^−1^, 142.1 to 154.3 mg L^−1^, and 104.5 to 128.6 mg L^−1^, respectively and, at the end of the experiment, it was found that 61.6%, 76.2%, and 64.3% of the total P in MCP, MS, and IS, respectively, was dissolved. At pH 7, O-P concentrations of MCP, MS, and IS were varied from 95.4 to 117.1 mg L^−1^, 135.9 to 152.5 mg L^−1^, and 90.1 to 112.9 mg L^−1^, respectively, and, at the end of the experiment, showed solubility of 58.5%, 76.2%, and 56.4%, respectively. Although the solubility of O-P from MCP was higher than MS at pH 2 and 4, no significant difference was observed (*p* > 0.05). However, the solubility of O-P from MS increased with the increase in pH and was found to be significantly different from MCP (*p* < 0.05). The in vitro solubility of O-P from IS was the lowest among the treatments and significantly different from MCP and MS in all the experiments (*p* < 0.05).

Animals are generally fed for higher performance, either in terms of reproduction or growth. Phosphorus plays a vital role in maintaining normal muscle growth, egg formation, and animal reproduction [41]. It also plays an important role in regulating acid-base and osmotic balance, energy and amino acid metabolism, and protein synthesis. Due to limited resources and high cost, nutritionists are trying to formulate diets considering the safety margins as well as dietary P requirements [42]. P solubility is one of the deterministic factors that influences the amount of supplement required for the animals. Factors including transit and retention time, pH, and particle size may control the P solubility in the gastrointestinal tract [43]. Moreover, P from inorganic sources are more soluble in the acid medium than the P from plant materials [44]. The amount of P available for absorption is controlled by its sustained solubility in the gastrointestinal tract in animals [45]. Primarily, P is absorbed in the small intestine within the pH range of 2 to 7 [46]. Studies reported that around 35% of the P is absorbed in the duodenum (pH 2 to 6.4), 5% in the jejunum (pH 6), and 40% in the ileum (pH 7) [47,48]. Struvite, an alternative P source has been extensively used as fertilizers in agriculture [13]; however, it is rarely tested as the P source in animal diets. As struvite is readily soluble under acidic conditions, the in vitro P solubility study was therefore conducted using pre-treated struvite as MS and IS by simulating the conditions in the gastrointestinal tract and compared with MCP. Among the test materials, P solubility from MS and MCP was not significantly different at a low pH. Moreover, due to an increase in P solubility from MS at comparatively higher pH (pH 5, 6, and 7), it can be said that, between the pre-treated struvites, MS could be better absorbed in the gastrointestinal tract. The solubility experiment results provide preliminary support of using MS as a potential alternative P source in animal diets. However, in vitro solubility testing has some limitations in extrapolating conditions in animals and further in vivo experiments are required for a better understanding of P metabolism.

## 4. Conclusions

In this study, neither any histopathological conditions in the internal organs nor any growth inhibition was found in rats fed with pre-treated struvites (MS and IS). The solubility test showed no significant difference in P solubility from MS and MCP at pH 2 and 4, while P solubility from MS increased at pH 5 to 7 and was found to be significantly different compared to MCP and IS. Considering resource recovery and recycling, efficacy as well as societal sustainability, struvite pre-treated as MS has the potential to be a sustainable alternative source of supplemental P in animal diets. Moreover, the results of this study motivate further studies with more stringent designs to better examine the potential of struvite application in diverse fields. Although commercialization of struvite may not be straightforward and is presumably dependent on circumstances such as legislation and national policies and economic feasibility in each country, P recycling is inevitable for both environmental protection and resource security in the world. 

## Figures and Tables

**Table 1:** Nutrient contents in the treatments.

Group	Treatment	Nutrient Content (mg kg^−1^)			
		P	N	Ca	Mg
I	DMSO (Control) ^a^	-	-	-	-
II	IS1 ^b^	0.26	0.00003	0.10	0.12
III	IS10 ^c^	2.60	0.0003	1.03	1.15
IV	MS1 ^d^	0.22	0.04	0.09	0.10
V	MS10 ^e^	2.21	0.37	0.88	0.98

^a^ DMSO = dimethyl sulfoxide; ^b^ IS1 = rats fed with IS at 1 mg kg^−1^ BW; ^c^ IS10 = rats fed with IS at 10 mg kg^−1^ BW; ^d^ MS1 = rats fed with MS at 1 mg kg^−1^ BW; ^e^ MS10 = rats fed with MS at 10 mg kg^−1^ BW.

**Table 2:** Chemical composition of recovered and pre-treated struvites and the highest standard limits of heavy metals in feedstock for animals, based on the Korean Regulatory Authority [27].

Parameters	Test Materials			Highest Standard Limits
	AS ^c^	MS ^b^	IS ^a^	
P (g kg^−1^ DM)	216	221	260	
Ca (g kg^−1^ DM)	85	88	103	
N (g kg^−1^ DM)	50	3713	0.03	
Mg (g kg^−1^ DM)	95	98	115	
K (mg kg^−1^ DM)	3567.6	NA ^e^	4407	-
Zn (mg kg^−1^ DM)	ND ^d^		ND ^d^	-
Ni (mg kg^−1^ DM)	ND ^d^		ND ^d^	-
Cu (mg kg^−1^ DM)	15.7		19.4	-
Cd (mg kg^−1^ DM)	ND ^d^		ND ^d^	1.0
Pb (mg kg^−1^ DM)	0.0001		0.0001	10.0
As (mg kg^−1^ DM)	0.0012		0.0015	2.0
Cr (mg kg^−1^ DM)	ND ^d^		ND ^d^	100.0
Hg (mg kg^−1^ DM)	ND ^d^		ND ^d^	0.4
Se (mg kg^−1^ DM)	ND ^d^		ND ^d^	2.0

^a^ AS = air-dried struvite; ^b^ MS = microwave irradiated struvite; ^c^ IS = incinerated struvite; ^d^ ND = not detected; ^e^ NA = not analyzed.

**Table 3:** P solubility from test materials at pH 2, 4, 5, 6, and 7 after four hours of incubation.

P Sources	P Solubility (%)				
	pH 2	pH 4	pH 5	pH 6	pH 7
MCP *	98.5 ± 0.7 ^a^	79.7 ± 0.1 ^a^	57.8 ± 3.7 ^a^	61.6 ± 0.5 ^a^	58.5 ± 0.5 ^a^
MS	83.4 ± 0.6 ^a^	77.2 ± 1.3 ^a^	78.6 ± 2.2 ^b^	76.2 ± 1.0 ^b^	74.8 ± 0.4 ^b^
IS	69.1 ± 2.6 ^b^	70.4 ± 0.3 ^b^	67.6 ± 1.0 ^c^	64.3 ± 0.6 ^c^	56.4 ± 0.2 ^c^

* MCP = Monocalcium phosphate; ^a,b,c^ Different superscript in the same column indicates statistical differences among the different treatment groups.
